# A Case of Entero-peritonitis with Suppuration

**Published:** 1877-08

**Authors:** Henry Van Buren

**Affiliations:** Chicago


### A CASE OF ENTERO-PERITONITIS WITH SUPPURATION.

By Dr. HENRY VAN BUREN, Chicago.

Willie S. Aged 4 years.

On the 21st of February last this little patient was attacked with vomiting—pulse, 120 per minute, and strong febrile symptoms.

On the 23d his pulse was 140, and the vomiting and fever continued.

The abdomen now became swollen, and the tongue coated and reddened at the edges and tip, and for one week all the symptoms of an ordinary case of enteritis were present.

On the 2d of March, the tympanitic character of the bowels had nearly disappeared, the other symptoms abated, and the patient was supposed to be convalescent.

On the 4th of March, I discovered the bowels swelling again; they were hard and tense, and pressure caused great pain. The pulse was 140, and all of the earlier symptoms were renewed. These continued and increased from the 4th to the 11th of March.

The abdomen was now distended to 36 in. in circumference, the normal size being about 18 inches, crowding the contents of the abdominal cavity against the diaphragm, and obstructing the respiration and the heart's action.

The veins over the abdomen became engorged, and could be readily traced over the whole surface.

The fæces were now dark and at times muco-sanguinolent, or purulent in character.

The patient on the 9th and 10th of March became greatly prostrated, partially comatose, and the vomiting continued troublesome.

The treatment thus far had been mostly opiates, with small doses of hydrarg. cum creta, and the diet beef tea with brandy.

Poultices were kept constantly over the bowels.

An effort was made to lessen the enormous distension of the bowels, by introducing a long flexible tube into the rectum and applying an air-pump. Some of the accumulated gases were thus removed.

On the evening of the 10th, the case of my little patient had become a desperate one. And, on a further examination of the abdomen, I discovered what I thought to be a cavity of pus, with the centre about one inch below the umbilicus, and its diameter probably not more than about 3 or 4 inches.

Dr. Norman Bridge had been in consultation with me during the progress of this case, and it was determined on the morning of the 11th of March to make an opening into the peritoneal cavity and intestines. Owing to uniform tympanitic resonance, the consulting physician did not believe that we would find pus, but that a puncture with an aspirating needle would be highly proper, to relieve the enormous distension of the bowels by the escape of gases. It has already been intimated that I believed there was pus, and as Dr. Bridge concurred in the opinion that an opening should be made, with his assistance I performed the operation.

The instrument used was a small trocar and canula, and the point of entrance made at about one inch below the umbilicus; the instrument was thrust into the abdomen a distance of about three inches. The trocar was withdrawn, and 20 ounces ot fluid escaped through the canula (which, under the microscope and by chemical test, proved to be pus with only slight traces of fecal matter).

On the afternoon of the same day, we made two additional openings with the needle of the aspirator, one on each side, a distance of about five inches from the umbilicus. This instrument was thrust into the intestines, and the pump applied, and through the aspirator we got a dark colored fecal discharge, but no pus.

On the following morning, ·March 12th, the opening first made with the trocar was slightly enlarged with a bistoury, and through this opening eighteen ounces of pus, additional to what had escaped—now discharged—some of which was lost by spurting out on the floor by the side of the puncture.

Incredible as it may appear, as shown above, no less than

38 ounces of pus, dense in quality, escaped through the opening made in the regio umbilicalis, within 36 hours after the operation.

The poultices were continued for one week, after which the abdomen was covered with cotton-batting. The opening remained for about three weeks, during which time, weak solutions of carbolic acid were injected into the opening.

The patient was given citrate of iron and quinine, brandy and lager beer, and took the extract of six pounds of beef every twenty-four hours.

We had in this case, I think, general enteritis, and circumscribed inflammation of the peritoneum, and the pus was found mostly in a well or pocket in the peritoneal cavity,in the region of the umbilicus.

I have this to say,respecting this little patient. His case is the most remarkable one of its character which I have seen on record, and it is wonderful that he has entirely recovered, and is to-day a healthy vigorous child.

Reported, July 16th, 1877.

To the Members of the Chicago Medical Society:—With the present issue, the first year of the publication of the Pathological Transactions of our Society, is completed. The net results of the undertaking up to this time, are the permanent record of twenty-one articles, some of which are novel, and all of which are of value to ourselves and the profession at large, and the creation of increased interest concerning pathology, on the part of our membership. But the Editor desires, in future, to interest a far larger proportion of the members of our Society, in the work of sustaining the publication of our "Transactions." Only fourteen of the members have contributed to the Transactions during the past year. A number quite too small, when compared with the roll of membership. Not one-half of the really valuable material presented to the Society during the past year, has been utilized for the benefit of science. For the future success of our enterprise, there should be, on the part of the members of the Society: 1st. A more careful and systematic observation of the course, progress, and results of interesting and unique cases. 2d. More general interest upon the subject of the pathological anatomy of fatal cases. 3d. The cultivation of the habit of reporting cases—especially those which terminate fatally—*in writing, to the Society*, so that such reports may be available for publication. The Editor of the Transactions respectfully urges that the foregoing hints be practically adopted by the members of the Society.

